# Identifying fallers among ophthalmic patients using classification tree methodology

**DOI:** 10.1371/journal.pone.0174083

**Published:** 2017-03-23

**Authors:** Paolo Melillo, Ada Orrico, Franco Chirico, Leandro Pecchia, Settimio Rossi, Francesco Testa, Francesca Simonelli

**Affiliations:** 1 Eye Clinic, Multidisciplinary Department of Medical, Surgical and Dental Sciences, University of Campania Luigi Vanvitelli, Naples, Italy; 2 School of Engineering, University of Warwick, Coventry, United Kingdom; Save Sight Institute, AUSTRALIA

## Abstract

**Purpose:**

To develop and validate a tool aiming to support ophthalmologists in identifying, during routine ophthalmologic visits, patients at higher risk of falling in the following year.

**Methods:**

A group of 141 subjects (age: 73.2 ± 11.4 years), recruited at our Eye Clinic, underwent a baseline ophthalmic examination and a standardized questionnaire, including lifestyles, general health, social engagement and eyesight problems. Moreover, visual disability was assessed by the Activity of Daily Vision Scale (ADVS). The subjects were followed up for 12 months in order to record prospective falls. A subject who reported at least one fall within one year from the baseline assessment was considered as faller, otherwise as non-faller. Different tree-based algorithms (i.e., C4.5, AdaBoost and Random Forests) were used to develop automatic classifiers and their performances were evaluated by the cross-validation approach.

**Results:**

Over the follow-up, 25 falls were referred by 13 patients. The logistic regression analysis showed the following variables as significant predictors of prospective falls: pseudophakia and use of prescribed eyeglasses as protective factors, recent worsening of visual acuity as risk factor. Random Forest ranked best corrected visual acuity, number of sleeping hours and job type as the most important features. Finally, AdaBoost enabled the identification of subjects at higher risk of falling in the following 12 months with a sensitivity rate of 69.2% and a specificity rate of 76.6%.

**Conclusions:**

The current study proposes a novel method, based on classification trees applied to self-reported factors and health information assessed by a standardized questionnaire during ophthalmological visits, to identify ophthalmic patients at higher risk of falling in the following 12 months. The findings of the current study pave the way to the validation of the proposed novel tool for fall risk screening on a larger cohort of patients with visual impairment referred to eye clinics.

## Introduction

Falls represent one of the most relevant public health problems for western countries, because of the implications for the quality of life and autonomy of elderly and their informal caretakers[1] and the related costs, estimated, on average, as about 9,000 euro to treat a fall [2].

Falls result from interactions of several risk factors, which have been distinguished in intrinsic (subject-based) and extrinsic (environmental or circumstantial) [3]. Among the hundreds of potential risk factors for falls which have been identified[4], several population-based studies have identified impaired vision as one of the most frequent risk factors for falls[5–8]: age-related macular degeneration, presbyopia, diabetic retinopathy, and glaucoma are responsible of various forms of visual impairments associated with increased fall risk[9]. Moreover, other visual factors, such as incorrect spectacle correction and use of multifocal glasses have also been linked with falls [9]. Finally, it has been suggested that some medications that can lead to ocular adverse events, e.g. antihistamines, antipsychotics, and tricyclic antidepressants, may contribute to falls by inducing changes in vision and vision loss[10]. Despite the evidences about the importance of visual assessment in patients who have fallen, and the strong attention for falls by the NHS in UK, a Royal College of Physicians audit in 2009 showed that most sites in UK did not employ a standardized visual acuity assessment. Moreover, most models developed to assess fall risk[11–18] are typically relying on performance-based physical assessment tasks, which are not usually performed in ophthalmologic clinics [17]. To the best of authors' knowledge, there is no specific tool designed to help ophthalmologists to screen visually impaired subjects for falling risk, during routine ophthalmologic visits. Conversely, as recently concluded in a literature review [19], understanding what components of vision are most likely to be responsible for increased fall risk, as well as other factors that can cause poor vision (e.g., incorrect prescription of lenses, not wearing glasses when needed, the inability to afford lenses, health issues such as diabetes, polypharmacy) need to be evaluated. For that reason, in our previous study, we developed a standardized questionnaire to collect information about the most relevant potential risk factors (about 80 variables) for falls, including lifestyles, general health, social engagement and eyesight problems, and data were collected from 150 ophthalmic patients [20]. Piloting this questionnaire in a retrospective study, we showed that tree-based classifiers enable the identification of patients with a previous history of falls with satisfactory sensitivity and specificity rates (>70% and >80%, respectively)[20]. These findings motivated the analysis of prospective 1-year follow-up data to assess the subjects who undergo at least one fall after the baseline assessment, as described in this manuscript.

The aim of the current study is to develop a tool to support ophthalmologists in identifying, among their patients, those exposed to the highest risk of falling in the twelve months after the clinical assessment, based on self-reported factors and health information assessed during standard ophthalmological visits. In order to build such a tool, beyond conventional statistical methods, such as logistic regression models and simple Bayesian classifier, we adopted more recent tree-based classification algorithms, such as C4.5[21], Random Forest [22] and Adaboost [23]. Our criteria for the best classification model is one that achieves satisfactory sensitivity and specificity rates in automatically identifying ophthalmic patients falling one year after the ocular visit, particularly, in comparison with performance of methods available in literature for fall risk assessment.

## Materials and methods

This study was conducted on a group of subjects aged 55 years and over, enrolled among the patients who attended the Eye Clinic of the “University of Campania Luigi Vanvitelli”, formerly named “Second University of Naples”, from February to July 2014. Two hundred subjects were enrolled into the first part of the study, which was to identify retrospective fallers and which was reported previously [20]. Those 200 subjects were invited to complete a follow-up prospective study, which is the currently reported study. Out of the 200 subjects originally enrolled, 141 completed the 12-month prospective follow-up study.

The research followed the tenets of the Declaration of Helsinki, and written informed consent was obtained before participant assessment. Ethics approval was obtained from the Institutional Review Board of the Second University of Naples.

Socio-demographic and medical data were recorded with a standardized questionnaire that was developed *ad hoc* and validated on retrospective data as detailed in our previous study [20]. Briefly, we selected variables, which have been investigated as potential risk factors for falls in previous studies, including but not limited to those on visual impaired subjects [4, 11, 16, 24–31]. The questionnaire, described in details in the S1 Table, is organized in four parts:

Part A, including health-related variables assessed by the ophthalmologist during the systemic anamnesis, e.g., number and type of prescribed drugs;

Part B, designed to include the relevant features about ocular conditions to be assessed during the eye visit, e.g., eye diseases, best corrected visual acuity (BCVA);

Part C, consisting of a self-administered questionnaire about lifestyle and social engagement;

Part D, consisting of a self-administered questionnaire to assess visual disability, based on Activity of Day Vision Scale (ADVS) scale (15 item version)[32].

The layouts of the self-administered parts of the questionnaire (i.e., Part C and D) are designed in order to be filled in by people with visual impairment, eventually with the support of their informal caretakers.

Finally, participants, eventually with the support of the informal caretakers, were asked to report any fall over a prospective 12-month follow-up, by filling in the provided calendar. A fall was defined as unintentionally coming to the ground or some lower level not as a result of a major intrinsic event (e.g., stroke) or overwhelming hazard (e.g., motor vehicle accident or violence). Therefore, participants were contacted monthly by telephone in order to record any falls experienced after the baseline visit. Consequently, those reporting at least one fall over the follow-up were labelled as fallers, the others as non-fallers.

### Statistical and data-mining methods

In order to assess that the two groups (fallers and non-fallers) presented no significant differences at the baseline, descriptive statistical analyses were performed and null hypothesis tested for each factor. Therefore, for each group all values of continuous and categorical variables were presented as mean ± standard error of the mean and as count and percentage, respectively. Unpaired t-tests were computed to compare continuous clinical variables, while chi-square tests were used to compare categorical variables between fallers and non-fallers. The Fisher exact test was used for categorical variables in case of a cell in the contingence 2 x 2 matrix with an expected frequency of less than 5. Moreover, we performed False Discovery Rate (FDR) analyses in order to adjust for the multiple testing problem[33].

In order to investigate the association between each risk factor at the baseline and the risk of falling in 12 months, binary logistic regression analysis with forward selection of variables was performed. At each step, the factor with the largest score statistic whose significance value was less than the default value of 0.05 entered the model, while factors with probability of a likelihood-ratio statistic based on the maximum partial likelihood estimates greater than 0.10 were left out of the models.

In order to develop a model to predict which patient would fall in the 12 months following the baseline assessment, different tree-based data-mining approaches were adopted: C4.5 [21], Random Forest [22] and AdaBoost [23]. Moreover, two benchmark classification algorithms, not based on decision trees, were adopted: Multinomial Logistic Regression model with a ridge estimator[34] and Naive Bayes (NB) classifier[35].

The decision tree algorithm C4.5 was developed by Quinlan et al.[21]. In the current study, it has been developed, using the J48 implementation (provided by the Weka data-mining tool), by varying the confidence factor (0.1 to 0.5 with step of 0.1). The algorithm was described in detail elsewhere[21].

Random Forest is the last tree-based classifier developed by Breiman[22]. In the current study, Random Forest was constructed using an ensemble of random trees from 10 to 40 with no depth limit and with the default number of randomly chosen features (i.e., log2(*n*)+1, where *n* is the total number of features). The algorithm is completely described in a study published by the developer [22].

AdaBoost is a widely used meta-learning algorithm, described in detail elsewhere [23]. In the current study, it was developed with the function AdaBoostM1, provided by the Weka data-mining tool; the simple tree classifier (i.e. decision stump) was adopted as weak classifier in the AdaBoost algorithm and the number of iteration was varied from 10 to 40.

Naïve Bayes is a classifier based on the Bayes theorem, described in detail elsewhere [35]. In the current study, the normal distribution kernel estimated was adopted without supervised discretization.

Multinomial Logistic Regression model with a ridge estimator is described in detail in the paper by leCessie and van Houwelingen [34]. We used the implementation provided by Weka (function Logistics) with default value of ridge estimator (i.e. 1.0E-8).

In order to assess the generation ability of the models, following the recommendation for tree-based classifier development in small datasets[36], we adopted the leave-one-out approach. According to this method, the classifiers were trained using the whole dataset except the data of one subject, and then, tested against the unseen (left-out) subject. The procedure was repeated for each subject and the performance measures were computed averaging the values among all the repetitions (in our study: 141). In the cross validation loop, we adopted a standard approach for learning from small and unbalanced datasets, based on oversampling that is the Synthetic Minority Over-sampling Technique (SMOTE)[37].

Receiver-Operator Characteristics (ROC) curves were constructed to compare the predictive value of each method for predicting prospective fall, and accuracy, sensitivity, specificity were computed according to standard formulae. Moreover, the diagnostic odds ratio was computed as a single indicator of diagnostic performance according to the following formula [38]:

$$diagnostic\;odds\;ratio=\frac{sensitivity\;x\;specificity}{\left(1-sensitivity\right)\;x\;\left(1-specificity\right)}$$

Statistical analysis was performed by employing the IBM SPSS Statistics (release 21.0.0.0, 2012; SPSS Inc., Chicago, IL). All the data-mining methods were implemented by the Weka platform for knowledge discovery (version 3.6.10), issued by the University of Waikato as an open source software under the GNU General Public License[39].

## Results

Among the 200 recruited subjects, 141 completed the 12-month follow-up (mean age, 73.2 ± 1.0 years), including 59 males (41.8%) and 82 females (58.2%). Participants had a range of severity of visual impairment, for example, BCVA ranging from no light perception to 20/20. 114 participants (80.9%) suffered from cataract in at least one eye, whereas 48 participants (34.0%) were pseudophakic in at least one eye. Moreover, 19 participants (13.5%) suffered from glaucoma and 16 (11.3%) showed age-related macular degeneration in at least one eye.

Over the 12-month prospective follow-up, 25 falls were referred by 13 patients: 5 patients reported one fall, 4 patients two falls, and 4 patients three falls. Descriptive statistical analyses for continuous and categorical variables, together with results of the statistical tests, are reported in S2 and S3 Tables, respectively. According to FDR analysis performed to adjust for multiple testing problem, none of the tests were significant.

The logistic regression analysis, reported in Table 1, showed that the following 3 variables were significantly associated with the risk of falling: “pseudophakia” and “use of prescribed eyeglasses” as protective factor (i.e., odds ratio lower than 1) and “recent worsening of visual acuity” as a predictive risk factor (i.e., odds ratio higher than 1). The classification table of the regression models against the whole dataset (i.e., resubstitution estimate) showed a high specificity rate (97.7%) even if with a low sensitivity rate of 30.8%.

**Table 1:** Logistic regression analysis for prospective falls with forward feature selection.

Features	Odds Ratio (95% CI)	p-value
Pseudophakia (The patient underwent cataract surgery for at least one eye at any time)	0.056 (0.006–0.550)	0.013
Use of prescribed eyeglasses (The patient used the eyeglasses prescribed by the ophthalmologist in the last visit)	0.094 (0.016–0.569)	0.010
Recent worsening of visual acuity (The patient complained a worsening of visual acuity in the last year)	6.120 (1.396–26.831)	0.016
Constant	0.169	<0.001

CI: confidence interval

**Fig 1:**
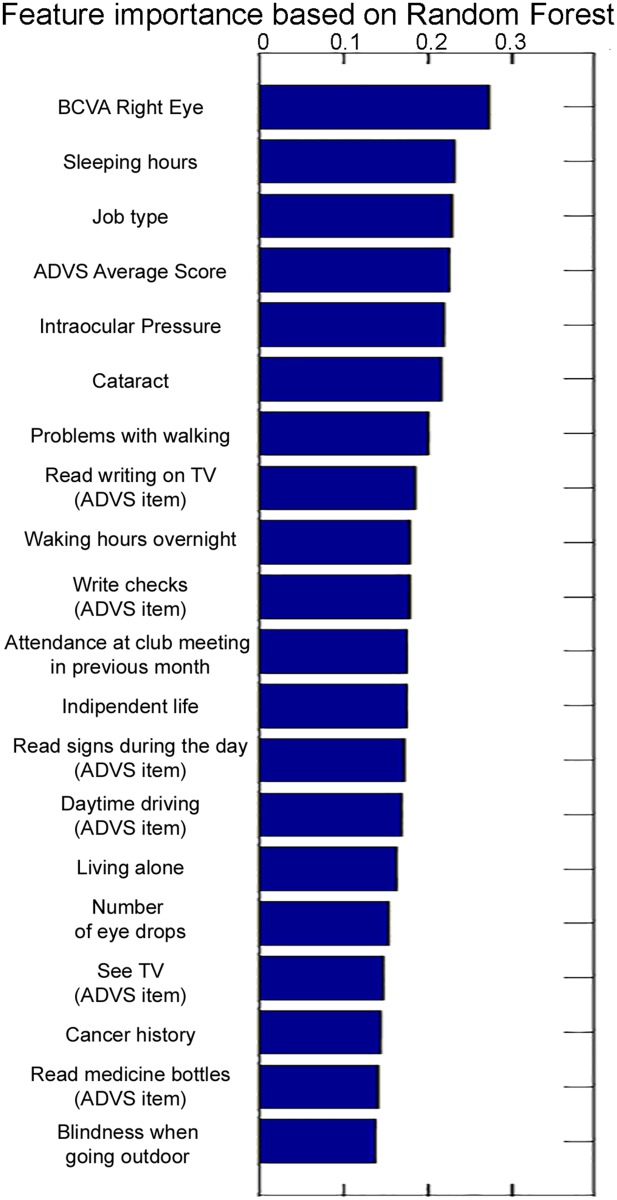
Plot of the importance values, estimated by the Random Forest algorithm, of the most relevant variables.

According to the data-mining Random Forest method, the most informative variables are: BCVA in the right eye, the number of sleeping hours and the type of job. As shown in Fig 1, reporting the feature importance of the 20 most relevant variables, seven were obtained by the ADVS questionnaire, i.e., the average score, and the following items: “Read writing on television”, “Write checks”, “Read signs during the day”, “Daytime driving”, “See television”, “Read medicine bottles”.

**Fig 2:**
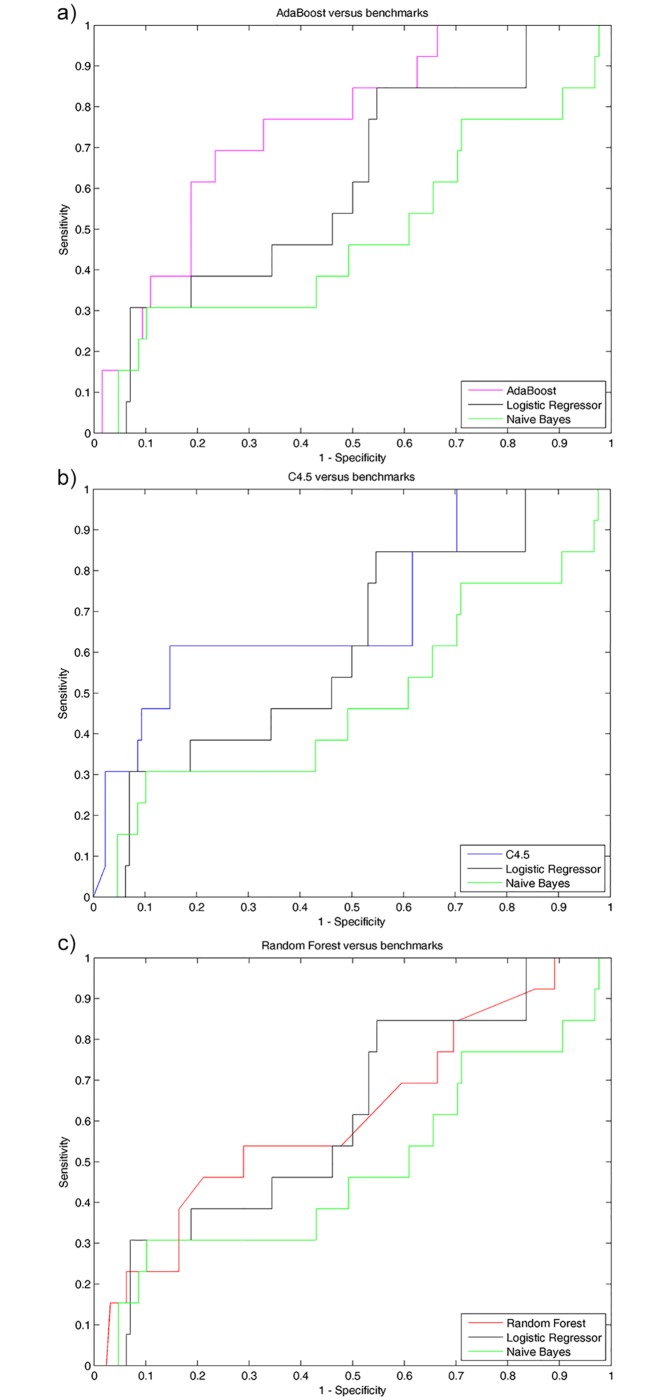
Comparison of ROC curves of the 3 tree-based classifiers, i.e., Adaboost (a), C4.5 (b) and Random Forest (c), compared to the two benchmark classifiers, developed to identify fallers among ophthalmic patients. Tree-based classifiers, particularly, AdaBoost outperformed the adopted conventional classification algorithm (i.e. Naïve bayesian classifier and logistic regression model).

Fig 2 shows a comparison of the ROC curves of the different adopted algorithms: the tree-based algorithms, particularly, AdaBoost achieved the largest Area under the Curve among the adopted classifiers, both conventional ones, i.e. Logistic Regression and Naïve Bayes classifier, and the other tree-based algorithm, i.e. Random Forest and C4.5. Moreover, the performance of the data-mining algorithms, in terms of binary classification measurement estimated by leave-one-out cross-validation, are reported in Table 2, in particular, AdaBoost achieved a sensitivity rate of 69.2% and a specificity rare of 76.6% with an overall accuracy rate of 75.9%.

**Table 2:** Comparison of the performance measures, estimated by cross-validation, of the best model for each classification tree algorithm.

	Accuracy	Sensitivity	Specificity	Area Under the Curve	Diagnostic odds ratio (95% CI)
AdaBoost	75.9%	69.2%	76.6%	75.1%	7.35 (2.11–25.57)
C4.5	83.0%	61.5%	85.2%	70.6%	9.18 (2.71–31.06)
Random Forest	69.5%	53.8%	71.1%	61.4%	2.87 (0.90–9.11)
Logistic regression	87.2%	30.8%	93.0%	60.6%	5.87 (1.51–22.87)
Naive Bayes	85.0%	23.1%	91.3%	48.2%	3.16 (0.76–13.23)

## Discussion

The research presented in this paper focused on the development of a novel tool, based on data-mining methods, designed to support ophthalmologists in recognizing, during ophthalmic visits, patients with visual impairment at higher risk of falling in the following 12 months in order to refer them to specialized fall clinics. This tool was designed to be used for patients before discharge from ophthalmological clinics, once their ophthalmological treatments are completed. Therefore, in this study, only clinical measures regularly used during ophthalmologic visits and patient self-reported information were employed.

In our previous study [20], after reviewing the literature on risk factors for falls, we developed a standardized questionnaire and tested its suitability to automatically detect fallers basing on retrospective data on self-reported falls in the 12 months preceding ophthalmologic visit. The present report proposes a tool, based on the above-cited questionnaire, that aimed to predict falls in the 12 months after an ophthalmologic visit. The current tool was validated in a sample of 141 ophthalmic patients that completed a 12 month follow-up, recruited from a single center (Eye Clinic of a University Hospital). We evaluated three machine learning techniques, particularly classification tree algorithms, as methods to identify ophthalmic patients at higher risk of prospective falls during the 12 months following the baseline examination. The application of these classification tree algorithms to develop predictive models proved to be advantageous for the type of research, such as fall risk investigation, which included explaining complex non-linear relationships and maximized the use of the data available. For example, the models based on tree classifiers achieved higher sensitivity rates than conventional classification models, such as Naïve Bayesian classifier and regression models. Moreover, we noticed that the variables selected as relevant factors by the binary logistic model (i.e, pseudophakia, use of prescribed eyeglasses, and recent worsening of visual acuity) are not included among the 20 most informative variables according to the feature importance estimated by Random Forest. This could be related to the differences in the methodology: logistic models use all (statistically significant) predictor variables simultaneously in the model, while classification tree algorithms use the predictor variables in a hierarchical and recursive manner; binary logistic models rely on data assumptions (i.e., the residuals associated with the logistic model are normally distributed), whereas classification tree algorithms do not assume any preliminary information regarding data distribution. Among classification tree algorithm, AdaBoost achieved higher performance than Random Forest and C4.5, in terms of area under the curve and sensitivity. We underline that logistic regression based on binary outcome (i.e., fallers versus non fallers), rather than linear regression modelling based on count data (i.e., number of falls), is adopted in order to have a classification model that could be more easily compared with the other data-mining methods.

Comparing with our previous study[20], based on the same potential risk factors but using as outcome the history of falls (i.e., any fall in the 12 months before the baseline assessment), although it is expected that some features could be more effective as predictor of prospective falls than indicator of past falls or vice-versa, we observed that three features (i.e., BCVA in the right eye, ADLS score and IOP) appear among the most six informative variables, according to Random Forest feature importance, in both studies, suggesting that these three features are good indicators of history of falls and predictors of prospective falls. Since the information provided by Random Forest may appear counter-intuitive with only the BCVA of the right eye being important, we performed an additional analysis including two features: BCVA in the better eye and BCVA in the worse eye. This analysis confirmed BCVA of the right eye among the most important features, while BCVA in the worse eye, in the better eye, and in the left eye ranked as 8^th^, 12^th^ and at 41^st^, respectively. Consequently, these results suggest that BCVA of the right eye is prevalent compared to BCVA in the left eye in our cohort. This could be explained by the fact that most Italian people are right-handed and consequently the right eye is dominant. Finally, the performances of the classifier developed in the previous study [20] are slightly higher than those achieved in the current study (e.g., AUC 86.2% versus 75.1%), in accordance with the statement by Gates et al. [40], that studies with a retrospective design may overestimate the accuracy of screening instruments.

The performances of the developed model are comparable with different fall risk assessment tools developed in general community-dwelling population proposed in literature[11, 15–18], mainly based on demographic information, self-reported problems with balance and coordination, as shown in Table 3. However, these models have been developed for clinicians caring for the general elderly population and not for ophthalmologists visiting visually impaired people. To the best of the authors’ knowledge, although several population-based studies have identified impaired vision as one of the most frequent risk factors for falls[5–8], only a few studies investigated on fall risk in visually impaired patients, particularly, age-related macular degeneration[30], glaucoma[41], or both[42] and no previous tool for fall risk assessment focused on ophthalmic patients. In particular, recently Boon et al.[42] investigated in a small size cohort (n = 80) over a short-term follow-up (8 weeks) whether several vision measures, including habitual binocular visual acuity, contrast sensitivity, visual fields, color vision, were predictive of incidence occurrence, including falls. Their binary logistic regression analysis showed contrast sensitivity as the only significant risk factor for fall prediction, whereas habitual binocular visual acuity and contrast sensitivity as significant factor for bumps. Although the performances of the model proposed by Boon et al. [42] (accuracy: 67.1%) are lower than ours (75.9%), we believe that these features, particularly, habitual binocular visual acuity should be considered in our future research activity, because we recorded that 84 participants needed to update their optical corrections in order to achieve BCVA. Unfortunately, according to the current study design, information regarding patient compliance is not available.

**Table 3:** Comparison of the performance of the model proposed in the current and previous studies to predict prospective falls among community-dwelling elderly.

Study	Test	Statistical method	Sen.	Spec.	AUC	Odds ratio (95% CI)
Current Study	Questionnaire + eye visit	Classification tree	69.2	76.6	75.1	7.35 (2.11–25.57)
Tromp, 2001[11]	Questionnaire	Multiple Logistic regression model	54	79	65	n/a
Russel, 2009[15]	Questionnaire	Multiple Logistic regression model	67.1	66.7	73	n/a
Bongue, 2011[16]	Questionnaire + one-leg balance test	Cox Regression Model	70.2	60.3	70.0	n/a
Gadkaree, 2015[17]	Questionnaire	Multivariate logistic regression model	n/a	n/a	69	n/a
Palumbo, 2015[18]	Questionnaire	Poisson Lasso regression model	n/a	n/a	63.9	n/a

Sen: Sensitivity; Spec.: Specificity; AUC: Area Under the Curve; n/a: not available

As regards the several tools for fall risk assessment proposed in literature, almost all of them require physical performance testing, which are not performed during standard ophthalmologic visits because they require specific clinical expertise, longer time compared to the proposed standardized questionnaire, and resource commitments[12]. On the contrary, the tool proposed in the present paper is feasible in any ophthalmologic clinic, since it only requires few ophthalmological parameters and the submission of a questionnaire, which could be easily performed in any clinic. For that reason, the proposed method could be used widely in inpatient ophthalmic settings to identify high-risk patients who could benefit from fall prevention programs[43] or fall detection system[44] and be referred to specialized fall-clinics. Finally, the tool has been designed to be integrated in a cloud-based platform[45], for risk assessment of vascular events[46] and falls. In particular, the current study was performed within the framework of a wider research activity focused on innovative methods for fall risk assessment in ophthalmic and cardiologic patients[47].

Our study has some limitations: the number of subjects used in this study is limited; the age of the sample is relatively young; contrast sensitivity, habitual binocular visual acuity, physical assessments of strength and balance were not performed in the study sample; and the validation in a larger, independent cohort is needed before these findings can be reliably generalized. In particular, the sample of the current study was taken from an eye clinic hospital population, which might have greater rates of abnormalities than the general population, limiting the applicability of our findings to non-hospital population.

Moreover, the major limitations of using data-mining methods is that the approach is not well known to ophthalmology, and its application in this field is relatively novel. Finally, the outcome variables (prospective fall over the one-year follow-up) were ascertained based on self-report, which may be subject to recall bias. However, we followed the recommendation Falls Network Europe Consensus[48] for fall recording by providing an *ad hoc* calendar for prospective fall recording and performing a monthly follow-up[48].

In summary, the current study showed that visual assessment and a standardized questionnaire, together with machine learning methods can be used in order to support ophthalmologists in screening their patients in regard to the risk of falling in 12 months and to refer those at higher risk to specialized fall clinics. The best machine-learning model, based on AdaBoost, enabled to identify fallers among ophthalmic patients with sensitivity and specificity rates of 69.2% and 76.6%, respectively. These findings pave the way to the validation of this novel tool for fall risk assessment on a larger cohort of elderly subjects.

## Supporting information

S1 Table List of all the variables investigated as potential factors related to falling risk. (DOCX)

S2 Table Comparison of continuous variables between fallers and non-fallers. (DOCX)

S3 Table Comparison of categorical variables between fallers and non-fallers. (DOCX)
