# Supplementary material for: Identifying fallers among ophthalmic patients using classification tree methodology
Source: PLoS One. 2017 Mar 23;12(3):e0174083. doi: 10.1371/journal.pone.0174083 (PMC5363841; doi:10.1371/journal.pone.0174083)
Supplement: S1 Table — (DOCX) [file pone.0174083.s001.docx]

| **S1 Table. List of all the variables investigated as potential factors related to falling risk** | | | |
| --- | --- | --- | --- |
| **Variables** | **Question** | **Categories or unit of measures** | **Ref.** |
| **Part A: Health-related variables assessed by the ophthalmologist during the systemic anamnesis** | | | |
| Gender | Abstracted by personal data records | male; female | [1-4] |
| Age | Abstracted by personal data records | Years |  |
| Weight | What's your weight? | Kg | [2, 3] |
| Height | How tall are you? | m^2^ |  |
| Body mass index | Computed from Weight and Height | Kg / m^2^ | [1, 4, 5] |
| Smoking habit | Do you smoke?  If not, did you ever smoke? | yes; no; in the past | [1-4] |
| Alcohol consumption | Do you consume alcohol? | Never; occasionally; usually; in the past | [1, 3] |
| Falls in the previous year | Did you fall during the last year? | Yes; no | [6] |
| Number of falls in the previous year | How many times did you fall the last year? | Integer (including 0) | [2] |
| Depression | Assessed by medical records,  evaluated by the physicians  and/or asked to the patients | Yes; no | [1, 6] |
| Anxiety |  | Yes; no | [4] |
| Urinary incontinency |  | Yes; no | [1, 6] |
| Osteoarthritis |  | Yes; no | [1, 2, 6] |
| Hypertension |  | Yes; no | [4] |
| Diabetes |  | Yes; no | [2, 4, 5] |
| Hearing loss and/or vestibular problems |  | Yes; no | [1, 5] |
| Cancer history |  | Yes; no | [4] |
| Parkinson disease |  | Yes; no | [4] |
| Alzheimer disease |  | Yes; no | [4] |
| Asthma |  | Yes; no | [4] |
| Cardiovascular disease |  | Yes; no | [4, 5] |
| Independent life | Are you independent in life activities? | Yes; no | [1] |
| Health compared with that of age group | How do you rate your health status  compared to people of your age? | Much healthier; healthier; as healthy as; less healthy; much less healthy | [3] |
| Shortage of breath | Do you suffer from shortage of breath? | No; yes; only if going uphill/hurrying | [7] |
| Problems with headaches | Do you have problems with headaches? | Yes; no | [7] |
| Problems with walking | Do you have problems with walking? | No problem; uses walking aid; gait problem (no aid); nonambulant | [8] |
| Sleeping hours | How long do you usually sleep overnight? | Hours | [7] |
| nocturnal awakenings | Do you wake up during the night? | Never; often; every night | [7] |
| waking hours overnight | How long are you awake overnight? | Hours | [7] |
| Number of prescribed drugs | Assessed by medical records and/or asked to the patients | Number of prescribed drugs | [1-4, 6, 8] |
| antidepressants |  | Yes; no |  |
| antipsychotics |  | Yes; no |  |
| antiemetic |  | Yes; no |  |
| sedatives and hypnotics |  | Yes; no |  |
| medicines for Parkinson's disease |  | Yes; no |  |
| antihypertensive or antiarrhythmic |  | Yes; no |  |
| analgesics |  | Yes; no |  |
| antiepileptic |  | Yes; no |  |
| Better vision | Do you see better during a sunny or a rainy day? | Sunny day; rainy day; indifferent | [9] |
| Blindness when going outdoor | Are you blinded because of broad daylight? | Yes; no | [9] |
| **Part B: Variables related to ocular conditions, abstracted from medical records and/or evaluated by the ophthalmologist during the eye visit** | | | |
| Cataract | Assessed whether the patient showed a cataract in at least one eye | Yes; no | [8, 10] |
| Pseudophakia | Assessed whether the patient underwent cataract surgery in at least one eye at any time | Yes; no |  |
| Glaucoma | Assessed whether the patient suffered from glaucoma in at least one eye | Yes; no |  |
| Age-related macular degeneration | Assessed whether the patient suffered from age-related macular degeneration in at least one eye | Yes; no |  |
| Other retinal degeneration | Assessed whether the patient suffered from other retinal degeneration in at least one eye | Yes; no |  |
| Use of bifocal / multifocal eyeglasses | Assessed whether the patient uses bifocal / multifocal eyeglasses | Yes; no | [3, 8] |
| Use of prescribed eyeglasses | Assessed whether the patient uses the lens prescribed by the ophthalmologist in the last visit | Yes; no | [3] |
| Use of eye drops | Assessed whether the patient uses eye drops | Yes; no | [9] |
| Number of eye drops | Assessed how many eye drops the patient uses | Integer number | [9] |
| Best corrected visual acuity (BCVA) in each eye | Measured during the ocular visits | Snellen decimal notation | [4, 5, 10] |
| Visual acuity loss within the last year in each eye | When previous measurements of BCVA (within the previous 12 months) are available demonstrating a BCVA worsening, the differences between the two measurements were computed | Snellen decimal notation | [8] |
| Recent worsening of visual acuity | Assessed whether the patient complained a worsening of visual acuity in the last year | Yes; no | [8] |
| Recent refraction change | Assessed whether the patient refraction changed in the last year | Yes; no | [8] |
| Intraocular pressure (IOP) | Measured during the ocular visit and the average value between the two eyes was computed | mmHg | [9] |
| **Part C: Self-administered questionnaire about the life style and social engagement** | | | |
| Living with | Do you live alone, with your spouse or with your family? | Alone; spouse; family | [1] |
| Type of house | What kind of house do you live in? | Condominium; single apartment | [1] |
| Job type | What is your job? | merchant or craftsman; worker; employed; freelancer; other | [1] |
| Retired | Are you retired? | Yes; no | [1, 11] |
| Frequency pushing/dragging heavy loads | How often do you push / drag heavy loads | Never; occasionally; 1 -2 per week; daily | [7] |
| Attendance at religious service in previous month | Have you attended religious service in the previous months? | Yes; no | [7] |
| Attendance at club meeting in previous month | Have you attended club meeting in the previous months? | Yes; no | [7] |
| Owns or cares for a pet | Do you own or care for a pet? | Yes; no | [7] |
| Contact with family/friends | Do you have sufficient contact with family / friends? | Sufficient; insufficient | [7] |
| Ability to raise € 350 in an emergency | Are you able to raise € 350 in an emergency | No difficulty; a little difficulty; lot of difficulty | [7] |
| **Part D: Self-administered questionnaire to assess visual disability, based on Activity of Day Vision Scale (ADVS) scale (15 item version)** | | | |
| Driving at night | Can you drive at night? | 5 - no difficulty; 4 – little difficulty;  3- moderate difficulty; 2- extreme difficulty;  1- unable because of poor vision;  Not Applicable (considered as missing data) | [12] |
| Seeing moving objects with night driving | Can you see moving objects with night driving? |  |  |
| Oncoming headlights | Can you see oncoming headlights? |  |  |
| Daytime driving | Can you drive during the day? |  |  |
| Drive in unfamiliar areas | Can you drive in unfamiliar areas? |  |  |
| Read signs at night | Can you read signs at night? |  |  |
| Read signs during the day | Can you read signs during the day? |  |  |
| See/recognize faces | Can you see / recognize faces at 4 meter distance? |  |  |
| Watch TV | Can you see television? |  |  |
| Read writing on TV | Can you read writing on television? |  |  |
| Read newspapers | Can you read newspapers? |  |  |
| Read medicine bottles | Can you read medicine bottles? |  |  |
| Read food cans | Can you read food cans? |  |  |
| Write checks | Can you write checks? |  |  |
| Thread a needle | Can you thread a needle? |  |  |
| ADVS average score | Computed as average of the scores of the above-mentioned items | 1 - 5 |  |

1. Bongue B, Dupre C, Beauchet O, Rossat A, Fantino B, Colvez A. A screening tool with five risk factors was developed for fall-risk prediction in community-dwelling elderly. J Clin Epidemiol. 2011;64(10):1152-60. Epub 2011/04/06. doi: 10.1016/j.jclinepi.2010.12.014. PubMed PMID: 21463927.

2. de Boer MR, Pluijm SM, Lips P, Moll AC, Völker‐Dieben HJ, Deeg DJ, et al. Different aspects of visual impairment as risk factors for falls and fractures in older men and women. J Bone Miner Res. 2004;19(9):1539-47.

3. Coleman AL, Stone K, Ewing SK, Nevitt M, Cummings S, Cauley JA, et al. Higher risk of multiple falls among elderly women who lose visual acuity. Ophthalmology. 2004;111(5):857-62. Epub 2004/05/04. doi: 10.1016/j.ophtha.2003.09.033. PubMed PMID: 15121359.

4. Knudtson MD, Klein BE, Klein R. Biomarkers of aging and falling: the Beaver Dam eye study. Arch Gerontol Geriatr. 2009;49(1):22-6. doi: 10.1016/j.archger.2008.04.006. PubMed PMID: 18513808; PubMed Central PMCID: PMC2703194.

5. Kulmala J, Era P, Parssinen O, Sakari R, Sipila S, Rantanen T, et al. Lowered vision as a risk factor for injurious accidents in older people. Aging Clin Exp Res. 2008;20(1):25-30. Epub 2008/02/20. doi: 4379 [pii]. PubMed PMID: 18283225.

6. Tromp AM, Pluijm SM, Smit JH, Deeg DJ, Bouter LM, Lips P. Fall-risk screening test: a prospective study on predictors for falls in community-dwelling elderly. J Clin Epidemiol. 2001;54(8):837-44. PubMed PMID: 11470394.

7. Bath PA, Pendleton N, Morgan K, Clague JE, Horan MA, Lucas SB. New approach to risk determination: Development of risk profile for new falls among community-dwelling older people by use of a genetic algorithm neural network (GANN). J Gerontol a-Biol. 2000;55(1):M17-M21. PubMed PMID: ISI:000088044100014.

8. Lord SR, Dayhew J, Howland A. Multifocal glasses impair edge-contrast sensitivity and depth perception and increase the risk of falls in older people. J Am Geriatr Soc. 2002;50(11):1760-6. Epub 2002/11/02. PubMed PMID: 12410892.

9. Melillo P, Orrico A, Attanasio M, Rossi S, Pecchia L, Chirico F, et al. A pilot study for development of a novel tool for clinical decision making to identify fallers among ophthalmic patients. BMC Medical Informatics and Decision Making. 2015;15(Suppl 3):S6. PubMed PMID: doi:10.1186/1472-6947-15-S3-S6.

10. Wood JM, Lacherez P, Black AA, Cole MH, Boon MY, Kerr GK. Risk of falls, injurious falls, and other injuries resulting from visual impairment among older adults with age-related macular degeneration. Invest Ophthalmol Vis Sci. 2011;52(8):5088-92. doi: 10.1167/iovs.10-6644. PubMed PMID: 21474773.

11. Ahmad R, Bath PA. The use of Cox regression and genetic algorithm (CoRGA) for identifying risk factors for mortality in older people. Health Informatics Journal. 2004;10(3):221-36. doi: 10.1177/1460458204042236.

12. Pesudovs K, Garamendi E, Keeves JP, Elliott DB. The Activities of Daily Vision Scale for cataract surgery outcomes: re-evaluating validity with Rasch analysis. Invest Ophthalmol Vis Sci. 2003;44(7):2892-9. PubMed PMID: 12824228.
