# Supplementary material for: Identifying fallers among ophthalmic patients using classification tree methodology
Source: PLoS One. 2017 Mar 23;12(3):e0174083. doi: 10.1371/journal.pone.0174083 (PMC5363841; doi:10.1371/journal.pone.0174083)
Supplement: S2 Table — (DOCX) [file pone.0174083.s002.docx]

**S2 Table. Comparison of continuous variables between fallers and non-fallers.**

| Feature | Non-faller subjects | Faller subjects | p-value | FDR-adjusted  p-value |
| --- | --- | --- | --- | --- |
| Age | 73.29±1.03 | 71.69±2.95 | 0.637 | 1.000 |
| Height | 164±0.72 | 165.38±3.55 | 0.581 | 1.000 |
| Weight | 72.38±1.06 | 73.23±2.11 | 0.801 | 1.000 |
| Body Mass Index | 26.89±0.35 | 26.91±0.73 | 0.959 | 1.000 |
| Number of falls in the previous year | 0.47±0.073 | 0.69±0.263 | 0.359 | 1.000 |
| Sleeping hours | 6.58±0.13 | 5.85±0.37 | 0.001 | 0.082 |
| Waking hours overnight | 0.9±0.13 | 1.31±0.5 | 0.002 | 0.082 |
| Number of prescribed drugs | 2.38±0.19 | 2.15±0.64 | 0.528 | 1.000 |
| Number of instilled eye drops | 0.22±0.05 | 0.31±0.17 | 0.563 | 1.000 |
| Best correct visual acuity Right Eye | 0.39±0.03 | 0.21±0.08 | 0.059 | 0.754 |
| Best correct visual acuity Left Eye | 0.4±0.03 | 0.28±0.09 | 0.196 | 1.000 |
| Visual acuity loss within the last year  in Right eye | 0.04±0.01 | 0.02±0.02 | 0.645 | 1.000 |
| Visual acuity loss within the last year  in Left Eye | 0.04±0.01 | 0.09±0.06 | 0.240 | 1.000 |
| Intraocular pressure | 15.81±0.21 | 14.38±0.43 | 0.129 | 0.882 |
| Driving at night | 3.7±0.15 | 3.77±0.32 | 0.875 | 1.000 |
| Seeing moving objects with night driving | 3.47±0.17 | 3.69±0.41 | 0.681 | 1.000 |
| Oncoming headlights | 3.38±0.18 | 3.62±0.42 | 0.675 | 1.000 |
| Daytime driving | 3.16±0.19 | 3.46±0.48 | 0.626 | 1.000 |
| Drive in unfamiliar areas | 3.36±0.17 | 3.46±0.48 | 0.858 | 1.000 |
| Read signs at night | 3.52±0.16 | 3.77±0.36 | 0.631 | 1.000 |
| Read signs during the day | 3.05±0.19 | 3.23±0.47 | 0.764 | 1.000 |
| See/recognize faces | 1.41±0.13 | 1.31±0.43 | 0.800 | 1.000 |
| See TV | 1.05±0.1 | 1.23±0.43 | 0.601 | 1.000 |
| Read writing on TV | 1.56±0.11 | 2±0.41 | 0.248 | 1.000 |
| Read newspapers | 1.94±0.15 | 2.15±0.58 | 0.666 | 1.000 |
| Read medicine bottles | 1.98±0.13 | 1.92±0.45 | 0.888 | 1.000 |
| Read food cans | 1.84±0.13 | 1.92±0.45 | 0.856 | 1.000 |
| Write checks | 1.66±0.15 | 2.08±0.57 | 0.415 | 1.000 |
| Thread a needle | 2.6±0.16 | 3±0.54 | 0.450 | 1.000 |
| ADVS Average score | 2.41±0.1 | 1.81±0.27 | 0.060 | 0.754 |

Details about the features, including units of measurement, are reported in S1 Table

FDR: False Detection Rate
