# Supplementary material for: Identifying fallers among ophthalmic patients using classification tree methodology
Source: PLoS One. 2017 Mar 23;12(3):e0174083. doi: 10.1371/journal.pone.0174083 (PMC5363841; doi:10.1371/journal.pone.0174083)
Supplement: S3 Table — (DOCX) [file pone.0174083.s003.docx]

**S3 Table. Comparison of categorical variables between fallers and non-fallers.**

| **Feature** | **Value** | **Low-risk subjects** | **High-risk subjects** | **p-value** | **FDR-adjusted**  **p-value** |
| --- | --- | --- | --- | --- | --- |
| Gender | Female | 76 (59.4) | 6 (46.2) | 0.357 | 1.000 |
|  | Male | 52 (40.6) | 7 (53.8) |  |  |
| Falls in the previous year | No | 88 (66.7) | 7 (53.8) | 0.275 | 1.000 |
|  | Yes | 44 (33.3) | 6 (46.2) |  |  |
| Smoking habits | In past | 27 (21.1) | 2 (15.4) | 0.767 | 1.000 |
|  | No | 72 (56.3) | 7 (53.8) |  |  |
|  | Yes | 29 (22.7) | 4 (30.8) |  |  |
| Alcohol consumption | In past | 3 (2.3) | 1 (7.7) | 0.562 | 1.000 |
|  | Never; | 72 (56.3) | 8 (61.5) |  |  |
|  | occasionally; | 7 (5.5) | 4 (30.8) |  |  |
|  | Usually | 46 (35.9) | 13 (100) |  |  |
| Depression | No | 110 (85.9) | 12 (92.3) | 1.000 | 1.000 |
|  | Yes | 18 (14.1) | 1 (7.7) |  |  |
| Anxiety | No | 84 (65.6) | 9 (69.2) | 1.000 | 1.000 |
|  | Yes | 44 (34.4) | 4 (30.8) |  |  |
| Urinary incontinency | No | 104 (81.3) | 11 (84.6) | 1.000 | 1.000 |
|  | Yes | 24 (18.8) | 2 (15.4) |  |  |
| Osteoarthitis | No | 50 (39.1) | 7 (53.8) | 0.377 | 1.000 |
|  | Yes | 78 (60.9) | 6 (46.2) |  |  |
| Hypertension | No | 40 (31.3) | 4 (30.8) | 1.000 | 1.000 |
|  | Yes | 88 (68.8) | 9 (69.2) |  |  |
| Diabetes | No | 90 (70.3) | 9 (69.2) | 1.000 | 1.000 |
|  | Yes | 38 (29.7) | 4 (30.8) |  |  |
| Hearing loss and/or vestibular problems | No | 90 (70.3) | 9 (69.2) | 1.000 | 1.000 |
|  | Yes | 38 (29.7) | 4 (30.8) |  |  |
| Cancer history | No | 113 (88.3) | 12 (92.3) | 1.000 | 1.000 |
|  | Yes | 15 (11.7) | 1 (7.7) |  |  |
| Parkinson disease | No | 128 (100) | 12 (92.3) | 0.092 | 0.754 |
|  | Yes | 0 (0) | 1 (7.7) |  |  |
| Alzheimer disease | No | 128 (100) | 13 (100) | 0.50 | 1.000 |
|  | Yes | 0 (0) | 0 (0) |  |  |
| Asthma | No | 122 (95.3) | 12 (92.3) | 0.50 | 1.000 |
|  | Yes | 6 (4.7) | 1 (7.7) |  |  |
| Cardiovascular disease | No | 78 (60.9) | 10 (76.9) | 0.205 | 1.000 |
|  | Yes | 50 (39.1) | 3 (23.1) |  |  |
| Independent life | No | 21 (16.4) | 3 (23.1) | 0.464 | 1.000 |
|  | Yes | 107 (83.6) | 10 (76.9) |  |  |
| Health compared with that of age group | Much healthier | 5 (3.9) | (0) | 0.379 | 1.000 |
|  | Healthier | 30 (23.4) | 2 (15.4) |  |  |
|  | As healthy as | 77 (60.2) | 7 (53.8) |  |  |
|  | Less healthy | 15 (11.7) | 4 (30.8) |  |  |
|  | Much less healthy | 1 (0.8) | (0) |  |  |
| Problems with headaches | No | 105 (82) | 11 (84.6) | 1.00 | 1.000 |
|  | Yes | 23 (18) | 2 (15.4) |  |  |
| Shortage of breath | No | 68 (53.1) | 10 (76.9) | 0.122 | 0.882 |
|  | Yes | 4 (3.1) | 1 (7.7) |  |  |
|  | only if going uphill/hurrying | 56 (43.8) | 2 (15.4) |  | 1.000 |
| Problems with Walking | Nonambulant | 2 (1.6) | 0 (0) | 0.931 | 1.000 |
|  | Uses walking aid | 23 (18) | 3 (23.1) |  |  |
|  | Gait problem  (no aid) | 13 (10.2) | 1 (7.7) |  |  |
|  | No problem | 90 (70.3) | 9 (69.2) |  |  |
| nocturnal awakenings | Never | 26 (20.3) | 4 (30.8) | 0.589 | 1.000 |
|  | Rarely | 54 (42.2) | 3 (23.1) |  |  |
|  | Always | 9 (7) | 1 (7.7) |  |  |
|  | Often | 39 (30.5) | 5 (38.5) |  |  |
| Antipsychotics | No | 126 (98.4) | 13 (100) | 1.000 | 1.000 |
|  | Yes | 2 (1.56) | 0 (0) |  |  |
| Antidepressants | No | 120 (93.7) | 12 (92.3) | 0.592 | 1.000 |
|  | Yes | 8 (6.3) | 1 (7.7) |  |  |
| Antiemetic | No | 125 (97.7) | 13 (100) | 1.000 | 1.000 |
|  | Yes | 3 (2.34) | 0 (0) |  |  |
| sedatives and hypnotics | No | 116 (90.6) | 12 (92.3) | 1.000 | 1.000 |
|  | Yes | 12 (9.38) | 1 (7.69) |  |  |
| medicines for Parkinson's disease | No | 128 (100) | 12 (92.31) | 0.092 | 0.754 |
|  | Yes | 0 (0) | 1 (7.69) |  |  |
| antihypertensive or antiarrhythmic | No | 42 (32.81) | 3 (23.08) | 0.551 | 1.000 |
|  | Yes | 86 (67.19) | 10 (76.92) |  |  |
| analgesics | No | 112 (87.5) | 12 (92.31) | 1.000 | 1.000 |
|  | Yes | 16 (12.5) | 1 (7.69) |  |  |
| Antiepileptic | No | 125 (97.7) | 13 (100) | 1.000 | 1.000 |
|  | Yes | 3 (2.34) | 0 (0) |  |  |
| Better vision in | Rainy day | 57 (44.53) | 6 (46.15) | 1.000 | 1.000 |
|  | Sunny day | 71 (55.47) | 7 (53.85) |  |  |
| Blindness when going outdoor | No | 54 (42.2) | 5 (38.5) | 1.000 | 1.000 |
|  | Yes | 74 (57.8) | 8 (61.5) |  |  |
| Cataract | No | 24 (18.75) | 3 (23.08) | 0.714 | 1.000 |
|  | Yes | 104 (81.25) | 10 (76.92) |  |  |
| Pseudophakia | No | 81 (63.28) | 12 (92.31) | 0.029 | 0.754 |
|  | Yes | 47 (36.72) | 1 (7.69) |  |  |
| Glaucoma | No | 113 (88.28) | 9 (69.23) | 0.077 | 0.754 |
|  | Yes | 15 (11.72) | 4 (30.77) |  |  |
| Age-related macular degeneration | No | 113 (88.28) | 12 (92.31) | 0.550 | 1.000 |
|  | Yes | 15 (11.72) | 1 (7.69) |  |  |
| Other retinal degeneration | No | 100 (78.13) | 9 (69.23) | 0.492 | 1.000 |
|  | Yes | 28 (21.88) | 4 (30.77) |  |  |
| Use of bi/multi-focal glasses | No | 118 (92.19) | 12 (92.31) | 0.732 | 1.000 |
|  | Yes | 10 (7.81) | 1 (7.69) |  |  |
| Use of prescribed glasses | No | 71 (55.47) | 11 (84.62) | 0.073 | 0.754 |
|  | Yes | 57 (44.53) | 2 (15.38) |  |  |
| Recent worsening of visual acuity | No | 88 (68.75) | 7 (53.85) | 0.353 | 1.000 |
|  | Yes | 40 (31.25) | 6 (46.15) |  |  |
| Recent refraction change | No | 123 (96.09) | 13 (100) | 1.000 | 1.000 |
|  | Yes | 5 (3.91) | 0 (0) |  |  |
| Use of eye drops | No | 105 (82.03) | 10 (76.92) | 0.443 | 1.000 |
|  | Yes | 23 (17.97) | 3 (23.08) |  |  |
| Living with | Alone | 28 (21.9) | 0 (0) | 0.167 | 1.000 |
|  | Spouse | 41 (32.0) | 5 (38.5) |  |  |
|  | Family | 59 (46.1) | 8 (61.5) |  |  |
| Type of house | Condominium | 101 (78.9) | 11 (84.6) | 0.863 | 1.000 |
|  | Single apartment | 26 (20.3) | 2 (15.4) |  |  |
| Job type | Merchant / craftsman | 7 (5.5) | 0(0) | 0.574 | 1.000 |
|  | Worker | 15 (11.7) | 1 (7.7) |  |  |
|  | Employed | 15 (11.7) | 3 (23.1) |  |  |
|  | Freelancer | 8 (6.3) | 2 (15.4) |  |  |
|  | Other | 81 (63.3) | 7 (53.8) |  |  |
| Retired | No | 39 (30.5) | 4 (30.8) | 0.950 | 1.000 |
|  | Yes | 88 (68.8) | 9 (69.2) |  |  |
| Frequency pushing/dragging heavy loads | Never | 57 (44.5) | 5 (38.5) | 0.217 | 1.000 |
|  | Occasionally | 56 (43.8) | 7 (53.8) |  |  |
|  | 1 -2 per week | 9 (7.0) | 0 (0) |  |  |
|  | Daily | 1 (0.8) | 1 (7.7) |  |  |
| Attendance at religious service in previous month | Yes | 72 (56.3) | 7 (53.8) | 0.890 | 1.000 |
|  | No | 42 (32.8) | 4 (30.8) |  |  |
| Attendance at club meeting in previous month | Yes | 36 (28.1) | 3 (23.1) | 1.000 | 1.000 |
|  | No | 92 (71.9) | 10 (76.9) |  |  |
| Owns or cares for a pet | Yes | 4 (3.1) | 2 (15.4) | 0.092 | 0.754 |
|  | No | 110 (85.9) | 9 (69.2) |  |  |
| Contact with family/friends | Sufficient | 24 (18.8) | 1 (7.7) | 0.579 | 1.000 |
|  | Insufficient | 90 (70.3) | 10 (76.9) |  |  |
| Ability to raise € 350 in an emergency | No difficulty | 108 (84.4) | 11 (84.6) | 0.846 | 1.000 |
|  | A little difficulty | 4 (3.1) | 0 (0) |  |  |
|  | Lot of difficulty | 14 (10.9) | 2 (15.4) |  |  |

FDR: False Detection Rate
